# The sonographic features of neonatal appendicitis: A case report

**DOI:** 10.1097/MD.0000000000008170

**Published:** 2017-11-10

**Authors:** Shu-Yu Si, Yi-Yi Guo, Jian-Feng Mu, Chao-Ying Yan

**Affiliations:** Department of Paediatrics, First Hospital of Jilin University, Changchun, P.R. China.

**Keywords:** abdominal ultrasound, neonatal appendicitis, ultrasounds

## Abstract

**Rationale:**

Neonatal appendicitis is extremely rare, and preoperative diagnosis is challenging. This study aimed to investigate the utility of ultrasound for the diagnosis of neonatal appendicitis.

**Patient concerns:**

Four cases of neonatal appendicitis were included in this case series. One was a female infant and the other 3 were male infants; they were aged from 10 to 17 days.

**Diagnoses:**

Neonatal appendicitis.

**Interventions:**

Four newborns in our hospital were diagnosed with neonatal appendicitis by abdominal ultrasound. Their sonographic features were summarized and compared with surgical and pathological findings.

**Outcomes:**

In these infants, abdominal ultrasound demonstrated ileocecal bowel dilatation, intestinal and bowel wall thickening, and localized encapsulated effusion in the right lower quadrant and the abscess area, which was assumed to surround the appendix.

**Lessons:**

Ultrasound is helpful for the diagnosis of neonatal appendicitis.

## 1 Introduction

Appendicitis is one of the most frequent causes of acute abdominal pain requiring surgery in children aged 5 to 12 years. However, neonatal appendicitis is extremely rare. Atypical clinical symptoms associated with appendicitis in the neonatal period make early diagnosis difficult. As neonates have low immunity, neonatal appendicitis is associated with high rates of perforation and mortality.^[1]^ A review of neonatal appendicitis cases reported in the English-language literature from 1901 to 2000 has been published.^[1]^ Since then, several new case studies of neonatal appendicitis have been described. These focused on patients’ clinical features, associated conditions,^[2–5]^ and the application of new treatment strategies.^[6]^ In recent years, ultrasound diagnostic techniques have played an increasingly crucial role in the diagnosis of appendicitis in children^[7]^; however, no reports have investigated the utility of ultrasound for the diagnosis of neonatal appendicitis. Herein, we report 4 cases of neonatal appendicitis that were diagnosed with the aid of abdominal ultrasound. We summarize, discuss, and compare their sonographic features with surgical and pathological findings.

### 1.1 Case report

The study protocol was approved by the ethical committee of the First Hospital of Jilin University. The medical records of the pediatric patients in our hospital who were diagnosed as neonatal appendicitis from September 2013 to February 2014 were retrospectively reviewed. The sonographic features of the patients were summarized. Written informed consent was obtained from patients’ parents/guardians.

Four cases of neonatal appendicitis were included in this case series. One was a female infant and the other 3 were male infants; they were aged from 10 to 17 days. Table 1 summarizes the demographic and clinical characteristics of the patients.

**Table 1:**
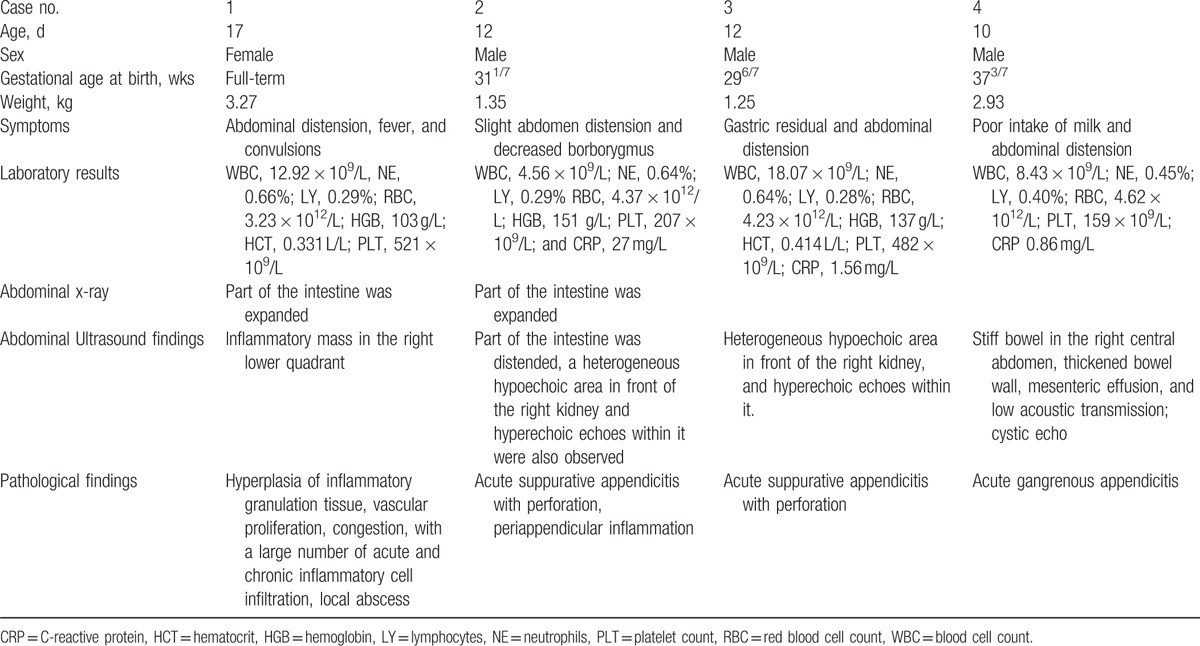
Neonatal appendicitis cases: summary data.

**Figure 1:**
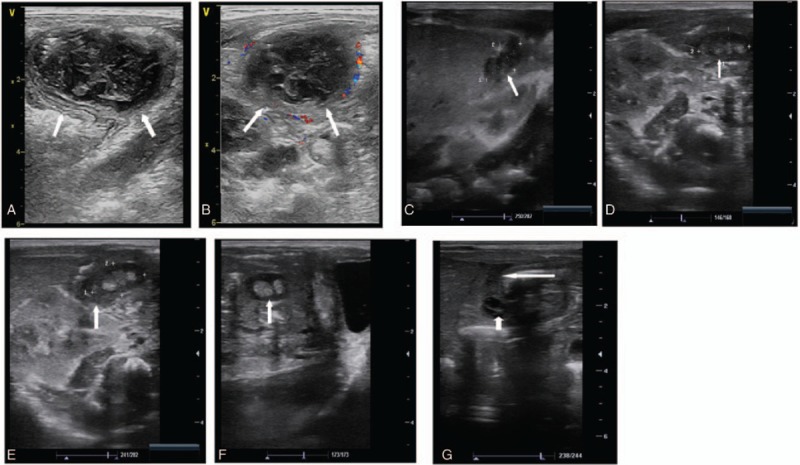
(A, B) A 17-day-old full-term female infant with abdominal distension, fever, and convulsions. Abdominal ultrasound shows a heterogeneous hypoechoic mass in the right lower quadrant (long- axis plane), 35 × 30 × 25 mm; and color doppler flow imaging shows a blood flow signal around the mass. (C, D) Preterm male infant with abdominal distension and tenderness in the right middle abdomen. Abdominal ultrasound shows a heterogeneous hypoechoic area in front of the right kidney and hyperechoic echoes within it. (E, F) Preterm male infant with gastric residuals and abdominal distension. Abdominal ultrasound shows a 14 × 7 mm heterogeneous echoic mass in the ileocecal area in the right lower quadrant with hyperechoic echoes inside. (G) A male infant in the neonatal intensive care unit with abdominal distension underwent abdominal ultrasound, which showed a stiff bowel in the right central abdomen, thickening of the bowel wall, mesenteric effusion, and low acoustic transmission (long arrow). A cystic echo was observed, which was considered an encapsulation (short arrow).

Case 1 was a 17-day-old full-term female infant. Abdominal ultrasound revealed a 35 × 30 × 25 mm heterogeneous hypoechoic mass in the right lower quadrant. There was hyperemia around the mass, and part of the intestine was stiff and fixed, with poor motility. The mass was considered an appendix abscess (Fig. 1A, B). Laparotomy was performed. Pathology demonstrated hyperplasia of inflammatory granulation tissue, vascular proliferation, congestion, a large amount of acute and chronic inflammatory cell infiltration, and a local abscess. After resection of the appendix, the patient recovered and was discharged 3 weeks after surgery.

Case 2 was a preterm male infant born at 31^1/7^ weeks of gestation. Abdominal ultrasound demonstrated that part of the intestine was distended. A heterogeneous hypoechoic area in front of the right kidney and hyperechoic echoes within it were also observed (Fig. 1C, D). Abdominal ultrasound suggested appendicitis. The right middle abdomen was painful to touch or pressure. Laparotomy was performed. Intraoperative findings showed a swollen appendix covered with pus and evidence of perforation. Appendectomy was performed. Postoperative diagnosis revealed acute suppurative appendicitis with perforation and periappendicular inflammation. The infant had a good recovery and was discharged after 4 weeks.

Case 3 was a preterm male infant. Abdominal ultrasound revealed an expanded and stiff ileocecum, thickening of the ileocecal wall in front of the right kidney, a heterogeneous echoic mass in the ileocecal area in the right lower quadrant, and hyperechoic echoes within it; the appendix was not visible (Fig. 1E, F). Abdominal ultrasound suggested appendicitis. Laparotomy was performed. Intraoperative findings showed a perforated appendix. Appendectomy was performed. Postoperative diagnosis revealed acute suppurative appendicitis with perforation. The infant recovered well after surgery.

Case 4 was a male infant delivered by cesarean section at 37^3/7^ weeks. Abdominal ultrasound (Fig. 1G) revealed a stiff bowel in the right central abdomen, thickening of the bowel wall, mesenteric effusion, and low acoustic transmission (long arrow). A cystic echo was observed; this was considered an encapsulation (short arrow). Surgical consultation found tenderness in the right abdomen, and did not exclude appendicitis. Laparotomy was performed. Intraoperative findings showed a perforated appendix. After resection of the appendix, the patient's postoperative status was good.

## 2 Discussion

Appendicitis is a common occurrence in childhood, but the diagnosis is extremely rare in neonates. Results of laboratory tests have less value for diagnosis in the neonatal period than for older patients,^[8]^ and clinical symptoms are atypical.

The 4 cases of neonatal appendicitis in this report showed no specific symptoms of neonatal appendicitis. They mainly presented with abdominal distension and anorexia. Three showed symptoms during the first 10 to 12 days after birth, while the exact time of symptom onset for the other case was not clear. All 4 cases had abdominal tenderness. Blood tests showed no obvious abnormalities, except for a slightly elevated white blood cell count in case 3, and increased levels of C-reactive protein in cases 2 and 4. Ultrasonography is more effective than plain radiography for depicting intraabdominal fluid, bowel wall thickness, and gut perfusion. Therefore, ultrasound may be useful for the diagnosis of neonatal appendicitis when associated with clinical manifestations. However, specific ultrasound diagnostic criteria for neonatal appendicitis have not been reported.

Current ultrasound diagnostic criteria for appendicitis are mostly relevant for children aged 5 to 12 years.^[9–11]^ In newborns, due to anatomical characteristics, inflammation and perforation of the appendix often occur rapidly. Diagnosis of neonatal appendicitis should not be excluded due to a lack of typical direct signs; indirect signs should also be noted. Findings from the 4 cases presented in this report and a literature review identified several key considerations for the diagnosis of neonatal appendicitis on ultrasound. Physicians should be aware that neonatal appendicitis is characterized by a vermiform appendix located in a high position, usually in the right abdomen or under the liver. However, newborns often suffer from perforated appendicitis, which can cause the appendix to be hard to visualize. In our 4 cases, we were only able to see an abscess located under the liver, which suggested a perforated appendix. Stiff and fixed intestines were observed in all 4 cases in this study. Importantly, ultrasound findings must be combined and interpreted with clinical manifestations.

## 3 Conclusion

The incidence of appendicitis is very low in newborns, who present with atypical symptoms, rapid disease progression, and frequent perforation and abscess. Abdominal ultrasound examination is recommended to confirm indications for surgery. If indications for surgery are present, we recommend immediate surgical treatment.
